# Assessment of Mumps Virus-Specific Antibodies: Comparison of Plaque Reduction Neutralization Test and Enzyme-Linked Immunosorbent Assay Estimates

**DOI:** 10.1093/infdis/jiz345

**Published:** 2019-07-11

**Authors:** Stéphanie Ravault, Damien Friel, Emmanuel Di Paolo, Adrian Caplanusi, Paul Gillard, Michael Povey, Stephane Carryn

**Affiliations:** 1 Clinical Laboratory Sciences, GSK, Rixensart; 2 R&D Center, GSK Vaccines, Wavre, Belgium

**Keywords:** mumps, vaccine, seropositive, seroresponse, plaque reduction neutralization test, enzyme-linked immunosorbent assay

## Abstract

**Background:**

The plaque reduction neutralization test (PRNT), which measures a subset of immunoglobulin antibodies (functional neutralizing antibodies), and the enzyme-linked immunosorbent assay (ELISA), which measures total immunoglobulin (neutralizing and nonneutralizing antibodies), characterize different aspects of the anti–mumps virus antibody response after vaccination.

**Methods:**

Data from a recent phase 3 clinical trial (NCT01681992) of 2 measles-mumps-rubella vaccines were used to compare anti-mumps antibody responses measured using an unenhanced PRNT (GSK; seropositivity cutoff and threshold, 2.5 and 4 times the 50% end-point dilution, respectively) with those estimated using an ELISA (thresholds, 5 and 10 ELISA units/mL, respectively).

**Results:**

Of 3990 initially seronegative samples, 3284 (82.3%) were seropositive after vaccination for anti-mumps antibodies in both assays. The Pearson correlation coefficient for double-positive samples was 0.57, indicative of a moderate correlation. Receiver operating characteristic curve analysis showed that an ELISA threshold of 51.7 ELISA units/mL best corresponded to the PRNT seroresponse threshold. There was no obvious vaccine brand effect on the correlation between assays.

**Conclusions:**

The moderate correlation between the anti-mumps antibody measurements obtained with PRNT and ELISA reflects different aspects of the serological response. In the absence of a well-defined protective serological threshold, PRNT provides complementary information on the antibody response, whereas ELISA remains a critically useful measurement of vaccine immunogenicity.

## Teaser

Data from a phase 3 clinical trial of measles-mumps-rubella vaccines show a moderate correlation between the anti-mumps antibody measurements derived by enzyme-linked immunosorbent assay and plaque reduction neutralization test, reflecting different aspects of the serological response measured by each assay.

There are no well-defined protective serological response threshold against mumps virus infection [1–4], and serological methods for detecting mumps antibodies have varying sensitivity and specificity [2]. Functional neutralizing antibody assays, such as the plaque reduction neutralization test (PRNT), are thought to provide the best estimate of protection [2], but there is no standard procedure for mumps virus infection [5].

The PRNT is cumbersome and labor intensive and therefore not readily amenable to high throughput, making it difficult to use in large-scale surveillance and vaccine trials [2]. In contrast, the enzyme-linked immunosorbent assay (ELISA) provides rapid, quantitative, reliably reproducible results, and is simple to perform. This has therefore become the most widely used technique in clinical serology testing, but it is not a functional assay because it cannot distinguish neutralizing from nonneutralizing antibodies.

GSK has developed an in-house PRNT that likely provides more discriminative titers and a more accurate measurement of immunogenicity to mumps vaccination than a previously used guinea pig complement- and anti-human immunoglobulin G (IgG)–enhanced PRNT [6]. The new assay determines neutralizing antibodies against the mumps wild-type strain Mu-90, which is considered better than using a vaccine-specific mumps strain when approximating the quality of vaccine-induced antibody to circulating wild-type strains [7].

Thresholds accepted by the Center for Biological Evaluation and Research within the US Food and Drug Administration, as defining clinically meaningful changes in anti-mumps antibody level following vaccination, were applied in analyses conducted in a recent phase 3 study. This trial assessed the immunogenicity and safety of measles-mumps-rubella (MMR) vaccines, MMR-RIT (Priorix; GSK) compared with MMR II (M-M-R II; Merck), when administered to toddlers; the clinical results are described elsewhere [8]. In the present study, serological data from the study are examined to compare anti-mumps virus antibody levels estimated using the PRNT with those measured using ELISA.

## METHODS

### Study Participants and Serum Samples

The serum samples used in this study were derived from a randomized, observer-blind study of the immunogenicity and safety of MMR vaccine formulations administered to 4516 children in their second year of life (NCT01681992), as described elsewhere [8]. The study was conducted in accordance with the Declaration of Helsinki and Good Clinical Practice guidelines and was approved by a national, regional, or investigational center institutional review board or independent ethics committee. Written informed consent was obtained from parents or legally acceptable representatives before enrollment. Participants were randomly assigned to receive MMR-RIT or MMR II, with the first dose administered at 12–15 months of age. The serum samples analyzed were from blood samples obtained before vaccination and 42 days after the first of the 2 MMR doses administered [8]. (To request access to patient-level data and documents for this study, please submit an enquiry via www.clinicalstudydatarequest.com.)

### Assays of Anti–Mumps Virus Antibody Levels

Concentrations of IgG antibodies to the mumps virus (Jeryl Lynn strain, genotype A) were measured using a whole-virus (wild-type strain) ELISA that was performed and interpreted as directed by the manufacturer (PPD). A PRNT without complement and without anti-IgG enhancement (unenhanced PRNT; GSK) [6], using the genotype D wild-type virus strain Mu-90 (MU90/Lo1), was used to assess the induction of functional neutralizing antibodies to the mumps virus. The unenhanced PRNT therefore used a more contemporary strain of mumps virus than that used in each MMR vaccine (RIT 4385 [Jeryl Lynn-derived strain] for MMR-RIT and Jeryl Lynn [homologous to ELISA antigen strain] for MMR II [8]). 

For the PRNT, briefly, 8 two-fold serial serum dilutions were made in 96-well plates and mixed with MU90/Lo1 strain. Susceptible Vero cells were then added to the serum-virus mixture to be infected by nonneutralized virus before forming a cell monolayer. After 2 days of incubation at 34°C and 5% carbon dioxide in a water-saturated incubator, the cell monolayer was fixed with ethanol/methanol. Viral antigens in infected cells were immunodetected with mouse anti-mumps monoclonal antibodies. After secondary labeling with anti-mouse horseradish peroxidase secondary antibodies (sheep anti-mouse IgG antibody conjugated to horseradish peroxidase [GE Healthcare; NA931-1]), a substrate (TrueBlue; KPL) was added for labeling, forming blue precipitates that allow direct counting of plaque-forming units in the well.

The ELISA and PRNT cutoffs for seropositivity and thresholds for seroresponse in children are shown in Table 1. The seropositivity cutoffs indicated the presence of anti-mumps antibodies. The seroresponse thresholds used in this study were determined to be statistically significantly different from the technical cutoffs of the assays, and they have been accepted by the Food and Drug Administration as thresholds defining active immunization offering clinical benefit.

**Table 1.** Anti-Mumps Antibody Seropositivity Cutoffs and Seroresponse Thresholds for Assays Tested

Assay	Seropositivity Cutoff	Seroresponse Threshold
ELISA concentration, EU/mL	5	10
PRNT titer, ED_50_	2.5	4

Abbreviations: ED_50_, 50% end-point dilution; ELISA, enzyme-linked immunosorbent assay; EU, ELISA units; PRNT, plaque reduction neutralization test.

### Statistical Analysis

The correlation between immune responses measured by each mumps assay was calculated using Pearson correlation coefficient on double-seropositive samples, where a value of 1 indicates total positive linear correlation and 0 indicates no linear correlation. A Deming regression analysis was also performed to quantify the relationship between ELISA and PRNT results. This was preferable to linear regression using ordinary least squares, because it considers the variability of both the dependent variable (log-transformed ELISA concentrations) and the explanatory variable (log-transformed PRNT titers). The Deming regression line was plotted over the scatterplot of log-transformed ELISA concentrations versus log-transformed PRNT titers, and 80% prediction interval limits were added.

A receiver operating characteristic (ROC) curve analysis was used to identify the ELISA seroresponse threshold that best corresponded to the PRNT seroresponse threshold of 4 times the 50% end-point dilution (ED_50_). Sensitivity was computed as the number of double-positive samples divided by the total number of PRNT-positive samples and specificity as the number of double-negative samples divided by the total number of PRNT-negative samples. These values were used to plot a ROC curve of sensitivity versus (1 − specificity). A perfect threshold would have sensitivity and specificity of 1; that is, all subjects with PRNT titers ≥4 ED_50_ would have ELISA concentrations above the threshold of 10 ELISA units [EU]/mL, and all subjects with PRNT titers <4 ED_50_ would have ELISA concentrations below the threshold of 10 EU/mL. The ELISA threshold that best corresponded to the PRNT seroresponse threshold had minimal distance from the perfect threshold marker in the top left corner of the ROC curve (sensitivity = 1 and 1 − specificity = 0), ie, the ELISA concentration that minimized the square root of [(1 − sensitivity)^2^ + (1 − specificity)^2^]. Statistical analyses were performed using SAS version 9.3 and SAS Drug Development 4.3 software.

## RESULTS

In the analysis of postvaccination seropositivity for antibodies to mumps antigen, of 3990 initially seronegative samples, 3284 were seropositive in both assays (ELISA, ≥5 EU/mL; PRNT, ≥2.5 ED_50_), 36 were seronegative in both, and 670 were discordant (16.8% of all samples; 664 ELISA positive and PRNT negative, 6 ELISA negative and PRNT positive). The correlation between immune responses measured by each mumps assay was calculated using the Pearson correlation coefficient, with the 3284 double-positive samples. This provided a value of 0.57, suggesting a moderate positive relationship.

**Figure 1.**
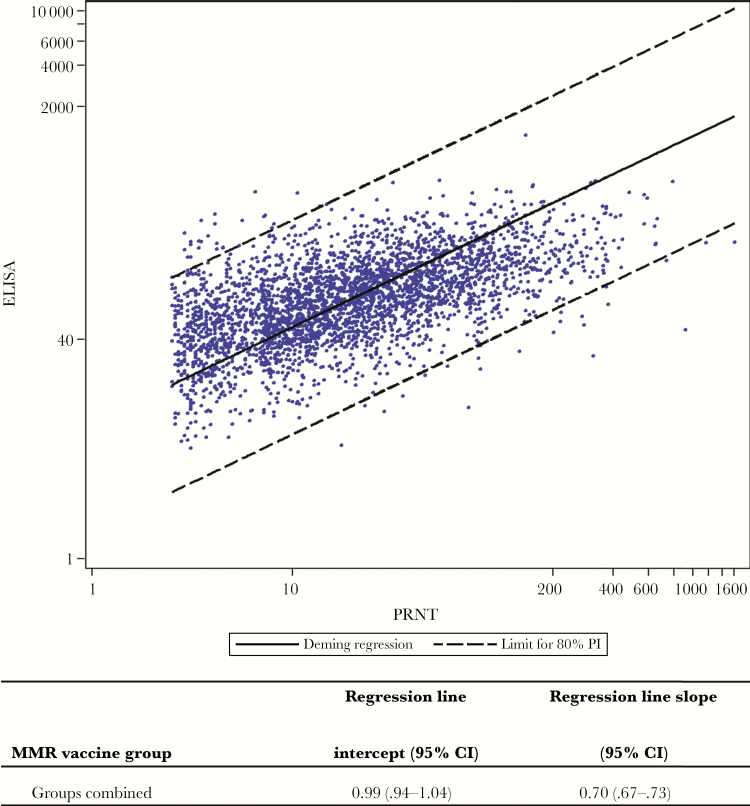
Scatterplot of mumps antibody values (log-transformed enzyme-linked immunosorbent assay [ELISA] concentrations versus log-transformed plaque reduction neutralization test [PRNT] titers), with Deming regression line and 80% prediction interval (PI) limits, in 3284 double-seropositive postvaccination serum samples. Abbreviations: CI, confidence interval; MMR, measles-mumps-rubella.

**Figure 2.**
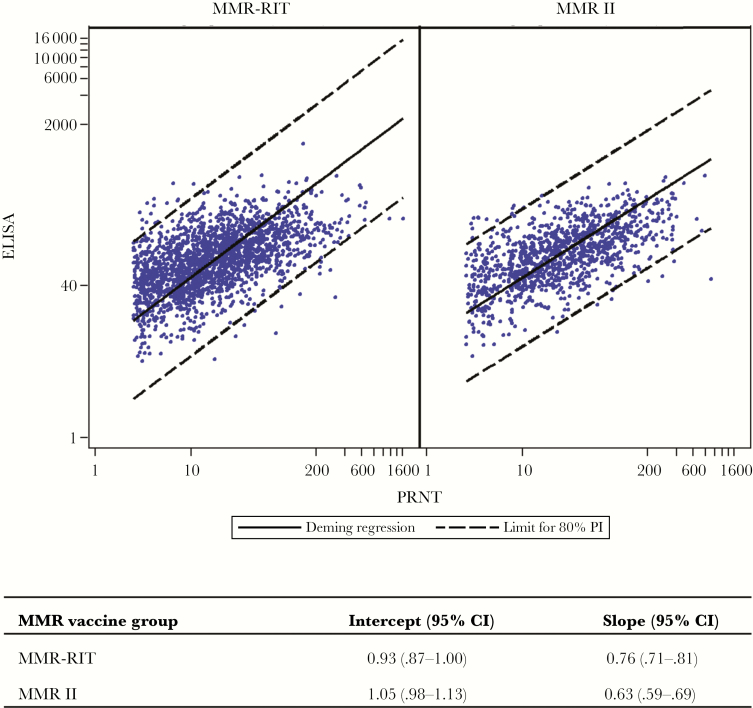
Scatterplot of mumps antibody values (log-transformed enzyme-linked immunosorbent assay [ELISA] concentrations vs log-transformed plaque reduction neutralization test [PRNT] titers), with Deming regression line and 80% prediction interval (PI) limits, in double-seropositive postvaccination serum samples, according to measles-mumps-rubella (MMR) vaccine received (MMR-RIT for 2107 serum samples; MMR II for 1177 serum samples). Abbreviation: CI, confidence interval.

Figure 1 shows a scatterplot of log-transformed postvaccination ELISA concentrations versus log-transformed PRNT titers from double-seropositive samples. Deming regression analysis of these data showed a slope of 0.70 (95% confidence interval, .67–.73) and intercept of 0.99 (.94–1.04), indicating a moderate correlation between the assays. Deming regression analysis of samples by MMR vaccine brand showed no obvious vaccine effect on the relationship between PRNT and ELISA (Figure 2).

**Figure 3.**
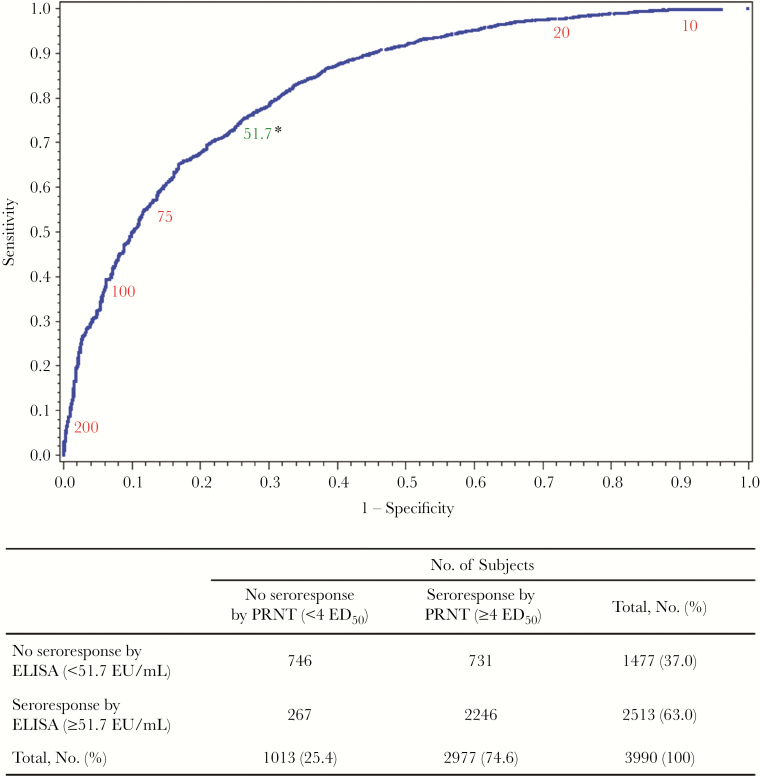
Receiver operating characteristic (ROC) curve of sensitivity (calculated as the number of double-positive samples divided by the total number of plaque reduction neutralization test [PRNT]–positive samples) versus “1 − specificity” (specificity calculated as the number of double-negative samples divided by the total number of PRNT-negative samples), using a PRNT seroresponse threshold of 4 times the 50% end-point dilution (ED_50_). Asterisk denotes the enzyme-linked immunosorbent assay (ELISA) threshold (in ELISA units per milliliter) best corresponding to the PRNT seroresponse threshold of 4 ED_50_.

An ROC curve sensitivity analysis was conducted to identify the ELISA seroresponse threshold that best corresponded to the PRNT seroresponse threshold of 4 ED_50_. The plot of assay sensitivity versus “1 − specificity” for postvaccination samples, using the PRNT seroresponse threshold of 4 ED_50_, is shown in Figure 3. An ELISA seroresponse threshold of 51.7 EU/mL was shown to be the optimal value, with maximized sensitivity and specificity, compared with the PRNT seroresponse threshold (Figure 3).

## Discussion

The new PRNT and used in this assessment is unenhanced, that is, without complement and anti-human IgG antibody enhancement. Because anti-IgG antibodies will recognize any IgG antibody (neutralizing or not) attached to the virus, an enhanced PRNT may provide a value closer to that expected from an ELISA that only measures neutralizing antibodies. Moreover, the unenhanced PRNT measures functional antibodies against mumps wild-type strain Mu-90, whereas the MMR-RIT vaccine strain is derived from the Jeryl Lynn strain, thus preventing bias in the measure, specifically overestimates [1], and providing better estimates of the quality of vaccine-induced antibody to the circulating wild type. The unenhanced PRNT therefore provides more discriminative titers and, hence, a more accurate estimate of immunogenicity to mumps vaccination than a previously used guinea pig complement– and anti-human IgG–enhanced PRNT [6]. The antigen used in the ELISA was whole virus (Jeryl Lynn strain, genotype A), which was homologous to the vaccine strain. Whole-virus ELISAs have been shown elsewhere to be more sensitive than protein antigen-based ELISAs, possibly because of the detection of antibodies against a wider range of proteins [9].

In our study, comparison of the unenhanced PRNT and the ELISA in the measurement of mumps virus-specific antibody levels indicates a moderate correlation, despite the fact that the assays measure different aspects of the serological response: the PRNT measures a subset of immunoglobulin antibodies (functional neutralizing antibodies), and the ELISA measures total immunoglobulin (neutralizing and nonneutralizing antibodies). Similar trends were noted in the results for all serum samples and according to the MMR vaccine received, MMR-RIT or MMR II. Overall, the rate of seropositivity was higher with the ELISA (3948 of 3990 samples, 98.9%) than with the PRNT (3290 of 3990 samples, 82.5%). As well as reflecting the different antibody populations measured by the assays, these results may have been influenced by the different mumps strain genotype used in each assay (wild-type strain Mu-90, genotype D, in the PRNT and Jeryl Lynn strain, genotype A, in the ELISA).

Differences in assay sensitivity may also have contributed toward the results. The PRNT threshold of 4 ED_50_ and ELISA threshold of 10 EU/mL correspond to seroresponse rates of approximately 80% and 98%, respectively [8]. Although the mechanism of protection against mumps infection is unknown and a protective serological response threshold is not defined [10, 11], these rates are in line with reported effectiveness rates for mumps vaccines [12]. In our ROC curve analysis, however, the PRNT threshold of 4 ED_50_ had the same seroresponse potential as an ELISA threshold of 51.7 EU/mL, which is considerably higher than the threshold of 10 EU/mL used in the clinical trial and suggestive of a large difference in assay sensitivity. 

A poor correlation between assays that measure mumps neutralizing antibody titers and other serologic methods, such as ELISA and immunofluorescent assays, has been reported elsewhere [2, 6, 13–20]. Differences have been shown in mumps antibody levels detected by antigen-specific ELISAs (against nucleoprotein, selected because of its immunodominance, and hemagglutinin, which has a role in viral neutralization) and PRNT [9]. This lack of correlation between the serological tests was thought to be due to differences in the antibody response to individual mumps proteins. The correlation found in our study was in line with a moderate correlation between the level of mumps-specific antibodies detected by fluorescent bead–based multiplex immunoassay and functional antibodies estimated by focus reduction neutralization test derived from mumps cases in the Netherlands [21].

A limitation of our analysis was that it was only based on serum samples obtained 42 days after the first MMR dose. In a 2-year follow-up study of children vaccinated at age 12–15 months with MMR-RIT or MMR II [22], the relationship between these 2 assays varied according to postdose timing, with an increase in PRNT titers between the 6-week and 1-year postvaccination time points, while ELISA antibody concentrations remained stable. This suggests maturation of the immune response, as shown by an increase in titers of functional antibodies measured by PRNT, after vaccination. Another limitation is that there may have been bias in determining the relative specificity or sensitivity of the assays because the PRNT and ELISA did not use the same mumps virus antigen genotype. Moreover, the seroresponse thresholds do not apply to anti-mumps antibody level estimates derived from other available ELISAs or neutralizing antibody tests. Finally, it should be considered that other immune mechanisms, including cellular responses, which are not reflected in the assays used in these studies, including our own, are likely to be involved in protection against mumps disease [23].

In conclusion, though there is a moderate correlation between the anti-mumps antibody measurements derived by ELISA and PRNT, the assays measure different aspects of the serological response. In the last decade, there have been several attempts to identify protective antibody titers [4, 7], but, despite some correlation between antibody titer and the likelihood of being protected, there is no well-defined protective serological threshold against mumps infection [10, 11]. Consequently, the ELISA remains a critically useful measurement of immunogenicity of vaccines, valid for between-vaccine comparisons and noninterference assessments. It is likely to be beneficial to complement these immune response assessments, in selected trials or populations, with the PRNT to obtain a more comprehensive evaluation of vaccine immunogenicity.

## Notes

***Acknowledgments.*** The authors acknowledge the major contribution of Rachel Collins, who died in early 2018, toward the initial development of this article. They thank Valérie Berthold and her laboratory team for ELISA data generation, and they also thank the Modis platform for editorial assistance and manuscript coordination, on behalf of GSK. Joanne Knowles (independent medical writer for Modis) provided medical writing support, and Giuseppe Chiapparo coordinated the manuscript development and editorial support.

***Author contribution.*** All authors had full access to all the data in the study, helped analyze or interpret the data, and take responsibility for the integrity of the data and the accuracy of the data analysis. All authors reviewed and commented critically on drafts of the manuscript for important intellectual content and gave final approval to submit the manuscript for publication. S. R. had the final responsibility to submit the manuscript for publication. Drafts were developed by a professional publication writer, according to the recommendations, documentation, and outline provided by the lead author (S. R.). S. R., P. G., M. P., and S. C. were involved in the conception or the design of the study. S. R., D. F., E. D. P., P. G., and M. P. participated in the collection or generation of the study data. A. C. performed the study. S. R. and E. D. P. contributed to the study with both the material and tools for analysis.

***Financial support.*** This work was supported by GlaxoSmithKline Biologicals, SA which was involved in all stages of the study conduct and analysis and covered the costs associated with developing and publishing the present article.

***Potential conflicts of interest.*** S. R., D. F., E. D. P., P. G., M. P., and S. C. are employed by the GSK group of companies, and A. C. is a former employee. S. R., E. D. P., D. F., P. G., and S. C. hold shares in the GSK group of companies as part of their employee remuneration. All authors have submitted the ICMJE Form for Disclosure of Potential Conflicts of Interest. Conflicts that the editors consider relevant to the content of the manuscript have been disclosed.

## References

[1] Rubin SA, Qi L, Audet SA, et al. Antibody induced by immunization with the Jeryl Lynn mumps vaccine strain effectively neutralizes a heterologous wild-type mumps virus associated with a large outbreak. J Infect Dis 2008; 198:508–15. 18558869 10.1086/590115

[2] Mauldin J, Carbone K, Hsu H, Yolken R, Rubin S Mumps virus-specific antibody titers from pre-vaccine era sera: comparison of the plaque reduction neutralization assay and enzyme immunoassays. J Clin Microbiol 2005; 43:4847–51. 16145156 10.1128/JCM.43.9.4847-4851.2005 PMC1234049

[3] Gouma S, Koopmans MP, van Binnendijk RS Mumps virus pathogenesis: insights and knowledge gaps. Hum Vaccin Immunother 2016; 12:3110–2. 27455055 10.1080/21645515.2016.1210745 PMC5215468

[4] Cortese MM, Barskey AE, Tegtmeier GE, et al. Mumps antibody levels among students before a mumps outbreak: in search of a correlate of immunity. J Infect Dis 2011; 204:1413–22. 21933874 10.1093/infdis/jir526

[5] Matsubara K, Fujino M, Takeuchi K, Iwata S, Nakayama T A new method for the detection of neutralizing antibodies against mumps virus. PLoS One 2013; 8:e65281. 23861738 10.1371/journal.pone.0065281 PMC3702533

[6] Pipkin PA, Afzal MA, Heath AB, Minor PD Assay of humoral immunity to mumps virus. J Virol Methods 1999; 79:219–25. 10381091 10.1016/s0166-0934(99)00019-1

[7] Gouma S, Ten Hulscher HI, Schurink-van ‘t Klooster TM, et al. Mumps-specific cross-neutralization by MMR vaccine-induced antibodies predicts protection against mumps virus infection. Vaccine 2016; 34:4166–71. 27372154 10.1016/j.vaccine.2016.06.063

[8] The MMR-161 Study Group. Immunogenicity and safety of measles-mumps-rubella vaccine at two different potency levels administered to healthy children aged 12–15 months: a phase III, randomized, non-inferiority trial. Vaccine 2018; 36:5781–8. 30104117 10.1016/j.vaccine.2018.07.076

[9] Latner DR, McGrew M, Williams NJ, Sowers SB, Bellini WJ, Hickman CJ Estimates of mumps seroprevalence may be influenced by antibody specificity and serologic method. Clin Vaccine Immunol 2014; 21:286–97. 24371258 10.1128/CVI.00621-13 PMC3957677

[10] McLean HQ, Hickman CJ, Seward JF The immunological basis for immunization series. Module 16: mumps https://apps.who.int/iris/bitstream/handle/10665/97885/9789241500661_eng.pdf?sequence=1. Accessed 28 May 2019.

[11] Plotkin SA Correlates of protection induced by vaccination. Clin Vaccine Immunol 2010; 17:1055–65. 20463105 10.1128/CVI.00131-10 PMC2897268

[12] Dayan GH, Rubin S Mumps outbreaks in vaccinated populations: are available mumps vaccines effective enough to prevent outbreaks? Clin Infect Dis 2008; 47:1458–67. 18959494 10.1086/591196

[13] Christenson B, Böttiger M Methods for screening the naturally acquired and vaccine-induced immunity to the mumps virus. Biologicals 1990; 18:213–9. 2257134 10.1016/1045-1056(90)90009-o

[14] Buynak EB, Whitman JE Jr, Roehm RR, Morton DH, Lampson GP, Hilleman MR Comparison of neutralization and hemagglutination-inhibition techniques for measuring mumps antibody. Proc Soc Exp Biol Med 1967; 125:1068–71. 6042404 10.3181/00379727-125-32278

[15] Berger R, Just M Comparison of five different tests for mumps antibodies. Infection 1980; 8:180–3.

[16] Backhouse JL, Gidding HF, McIntyre PB, Gilbert GL Evaluation of two enzyme immunoassays for detection of immunoglobulin G antibodies to mumps virus. Clin Vaccine Immunol 2006; 13:764–7. 16829613 10.1128/CDLI.00199-05 PMC1489562

[17] Date AA, Kyaw MH, Rue AM, et al. Long-term persistence of mumps antibody after receipt of 2 measles-mumps-rubella (MMR) vaccinations and antibody response after a third MMR vaccination among a university population. J Infect Dis 2008; 197:1662–8. 18419346 10.1086/588197 PMC9169514

[18] Matsubara K, Iwata S, Nakayama T Antibodies against mumps virus component proteins. J Infect Chemother 2012; 18:466–71. 22215227 10.1007/s10156-011-0358-3

[19] Allwinn R, Zeidler B, Steinhagen K, et al. Assessment of mumps virus-specific antibodies by different serological assays: which test correlates best with mumps immunity? Eur J Clin Microbiol Infect Dis 2011; 30:1223–8. 21455663 10.1007/s10096-011-1216-z

[20] Linde GA, Granström M, Orvell C Immunoglobulin class and immunoglobulin G subclass enzyme-linked immunosorbent assays compared with microneutralization assay for serodiagnosis of mumps infection and determination of immunity. J Clin Microbiol 1987; 25:1653–8. 3654938 10.1128/jcm.25.9.1653-1658.1987 PMC269301

[21] Kaaijk P, Gouma S, Hulscher HI, et al. Dynamics of the serologic response in vaccinated and unvaccinated mumps cases during an epidemic. Hum Vaccin Immunother 2015; 11:1754–61. 26047038 10.1080/21645515.2015.1040967 PMC4514281

[22] Berry AA, Abu-Elyazeed R, Diaz-Perez C, et al. Two-year antibody persistence in children vaccinated at 12-15 months with a measles-mumps-rubella virus vaccine without human serum albumin. Hum Vaccin Immunother 2017; 13:1516–22. 28481690 10.1080/21645515.2017.1309486 PMC5512763

[23] Ramanathan R, Voigt EA, Kennedy RB, Poland GA Knowledge gaps persist and hinder progress in eliminating mumps. Vaccine 2018; 36:3721–6. 29784466 10.1016/j.vaccine.2018.05.067 PMC6031229

